# Nano-Biosensing Platforms for Detection of Cow’s Milk Allergens: An Overview

**DOI:** 10.3390/s20010032

**Published:** 2019-12-19

**Authors:** Monika Nehra, Mariagrazia Lettieri, Neeraj Dilbaghi, Sandeep Kumar, Giovanna Marrazza

**Affiliations:** 1 Department of Bio and Nano Technology, Guru Jambheshwar University of Science and Technology, Hisar-Haryana 125001, India; ssmonikanehra@gmail.com (M.N.);; 2 Department of Chemistry “Ugo Schiff”, University of Florence, Via della Lastruccia, 350019 Sesto Fiorentino (Fi), Italy; mariagrazia.lettieri@unifi.it

**Keywords:** allergens, immunosensors, aptasensor, electrochemical sensor, optical sensor

## Abstract

Among prevalent food allergies, cow milk allergy (CMA) is most common and may persist throughout the life. The allergic individuals are exposed to a constant threat due to milk proteins’ presence in uncounted food products like yogurt, cheese, and bakery items. The problem can be more severe due to cross-reactivity of the milk allergens in the food products due to homologous milk proteins of diverse species. This problem can be overcome by proper and reliable food labeling in order to ensure the life quality of allergic persons. Therefore, highly sensitive and accurate analytical techniques should be developed to detect the food allergens. Here, significant research advances in biosensors (specifically immunosensors and aptasensors) are reviewed for detection of the milk allergens. Different allergic proteins of cow milk are described here along with the analytical standard methods for their detection. Additionally, the commercial status of biosensors is also discussed in comparison to conventional techniques like enzyme-linked immunosorbent assay (ELISA). The development of novel biosensing mechanisms/kits for milk allergens detection is imperative from the perspective of enforcement of labeling regulations and directives keeping in view the sensitive individuals.

## 1. Introduction 

In recent decades, the increased prevalence of food allergy has posed challenges to both food industry and clinical allergology. The European Academy of Allergology and Clinical Immunology (EAACI) has defined the term ‘food allergy’ [1]. Food allergy can be defined as abnormal immune system reaction after eating a certain food. The specific components of the food are responsible for these allergic reactions in sensitized subjects. Adverse food reactions can be categorized into toxic as well as non-toxic reactions, where non-toxic reactions are non-immune-mediated reactions and more common than toxic reactions. These can involve reaction to particular substances (like intolerance to lactose) as well as enzyme defects (like vasoactive amines). The immune-mediated reactions are affecting millions of people in terms of life-threatening reactions. Food allergy is exclusively due to immunoglobulin E (IgE)-mediated reactions. The involved food does not necessarily adversely affect the all non-allergic individuals. The food allergy symptoms can be observed from skin rashes (like mild urticaria) to life-threatening reaction (like anaphylactic shock) [2]. In the Western world, diagnosed food allergies affect approximately 1–2% of the total population. The greatest prevalence of food allergy, up to 8%, has been reported in younger children (<3 years of age). The complete and official list of allergens has also been provided by the World Allergy Organization (WAO) [3].

Cow’s milk allergy (CMA) has been identified as one of the most common and first cause of food allergy during early childhood because cow’s milk is the first food introduced into the diet of an infant. After peanut and tree nuts, milk allergy is the third most common allergy among the eight major food allergens. It accounts for approximately 20% of all food-induced anaphylactic reactions based on a study in United States [4]. 

The CMA prevalence varies depending on different approaches that are used for diagnosis, geographical factor, and age differences in the studied population. As per the reports, CMA has been identified in <0.5% of adults, 0.3% of older children, and 0.6–2.5% of preschoolers [5]. Over the past decade, the increasing incidences of worldwide CMA have been found for which the reasons are still unclear. The hypothesis behind these increased incidences has been reported in terms of hypersensitivities linked with increased cases of infant feeding with cow’s milk over breast-feeding [6].

The persistence of CMA during adult life can force an individual for complete elimination of cow’s milk from diet, since there is no treatment for CMA. However, the absence of milk in the diet can cause nutritional deficiency along with adverse effects over the growth of infants and/or children. The most common strategy to overcome this issue is to destroy/modify the structure of the milk allergens via food processing (i.e., heat treatment, enzymatic hydrolysis, fermentation, etc.) [7]. Cow’s milk has more than 25 different proteins. The concentration of proteins in milk is around 30–35 grams per liter [8]. Out of these proteins, only a few have been identified to be allergenic for which the immune system reacts abnormally.

Currently, allergens identification has gained significant research interest due to increasing information about the sequence and structure of allergens. This information has permitted the development of databases (i.e., Allergome) [9] that provide biochemical, clinical, and molecular data of several allergens. Different milk allergen proteins and their characteristics are listed in Table 1. In particular, two fractions of raw skim milk can be produced through acidification at pH 4.6 and 20 °C such as: The coagulum that contains the casein proteins (i.e., 80% of the total milk proteins), and the lactoserum that contains whey proteins (i.e., 20% of the total milk proteins) [10,11]. 

Casein proteins are the most abundant in the milk but there are lot of individuals with CMA induced by whey proteins [7]. The several protein variants can be easily degraded by proteolytic enzymes during digestion. The casein fraction precipitates in defined condition and consists of four different proteins with different percentages of the whole fraction such as (i) αS1-casein (32%), (ii) αS2-casein (10%), (iii) β-casein (28%), and (iv) κ-casein (10%). Here, αS1-casein has been found to be the most significant allergen of casein fraction [13]. 

Allergens of the whey fraction include (i) α-lactalbumin (α-LA), (ii) β-lactoglobulin (β-LG), (iii) immunoglobulins (Ig), (iv) bovine serum albumin (BSA), and (v) traces of lactoferrin [8]. The whey proteins own tertiary, secondary, and quaternary structures (especially in case of β-LG). The presence of intramolecular disulfide bonds in whey proteins stabilize their structure; the 3D structure contributes to the allergenicity of the protein due to its importance in preserving the conformational epitopes. Moreover, the whey proteins are not phosphorylated proteins. Among these, the most allergenic proteins are β-LG (50% of whey fraction) and α-LA (25% of whey fraction).

β-LG is the main cow milk whey protein. It can also be found in milk of other mammalian species but not in human and rodent’s milk. β-LG, being a small protein, possesses a molecular weight of 18.3 kDa and also antioxidant activity. It consists of 162 amino acids (Table 1). It is associated with the lipocalin family, playing an important role in the transportation of small hydrophobic molecules such as cholesterol and vitamin D2 [14,15]. It also binds Fe^2+^ and Cu^2+^ ions along with hydrophobic ligands (such as retinol). There are two different genetic forms of β-LG: β-lactoglobulin A (β-LGA) and β-lactoglobulin B (β-LGB). The difference between these two forms of β-LG depends upon amino acids, i.e., Asp-64 and Val-118 in β-LGA, which are replaced by glycine and alanine, respectively, in β-LGB [16]. β-LG can form long stiff fibers under continuous heating at low ionic strength as well as low pH. Under these conditions, β-LG solution forms a gel with a destabilized structure. The accountability of two disulphide bonds has been reported for high stability against acidic hydrolysis and proteases [8]. This property of β-LG enables preserving the structural integrity after digestion that allows its absorption via intestinal mucosa as well as further presentation to immunocompetent cells with high allergenic potential. This protein is majorly responsible for food allergy in approximately 60 to 80% of CMA patients [7]. 

The allergenic potential of α-LA (Bos d 4) is due to the presence of a lot of epitopes, which selectivity bind IgE [7]. α-LA is responsible for the lactose synthesis and also owns high stability due to the presence of four disulfide bridges and a high-affinity binding site for calcium that helps in stabilization of its secondary structure. BSA is present in small amounts in milk but nevertheless is an allergenic protein. Further, the allergenicity of BSA is due to the presence of the disulfide bridges that maintain the native antigenic determinants. Its biological role can be viewed in the transport, metabolism, several substances distribution, and further protection from free radicals [17]. 

The lactoferrin, a protein in the family of transferrin, is a glycoprotein. It is present in low quantities (<1%) in milk of most of the species [18]. It functions as an antioxidant and free radicals’ scavenger as well as defend the organisms against infections and inflammations that can be attributed to its ability of iron sequester from the environment [19]. Besides its involvement in detoxification processes, its antineoplastic effect is important in inhibiting the attachment of tumor growth factors. The allergenic activity of this protein is still not clear as the presence of IgE against other major milk allergens can also be found in milk-allergic individuals possessing lactoferrin-specific IgE [7,20].

The confusion of milk allergy with milk intolerance is common due to their similar symptoms. In comparison to allergy, the milk intolerance is not so dangerous; it can be specified as non-immunological reaction to certain component of milk that cause disorders in absorption, metabolism, or digestion. In case of milk allergy, the IgE-associated symptoms (relating to skin, respiratory system, and gastrointestinal tract) can be seen immediately or in sometime (about 2 h) after milk intake. Sometimes, complex and systematic anaphylactic response can be observed due to involvement of one or more target organs [21,22]. Different standard gold diagnosis methods for CMA are available such as the double-blind, placebo-controlled oral food challenge (DBPCFC) and unblinded oral food challenge (OFC) [23]. Besides less rigoristic nature and validation of OFC tests, both tests suffer from several limitations in terms of inherent risk of anaphylaxis, time-/resource-intensive nature, inappropriate for clinical practice (especially in large epidemiologic studies), and also safety concerns in atopic children. 

This review presents development of aptamers, biosensors, aptasensors for determination of allergen concentration in food matrix. The existing reviews mainly cover development of aptamers for allergens [24], biosensors for food allergens [25], confirmatory methods for allergens [26], and aptasensors for food analysis [27]. The present review is specifically designed to consider the cow milk allergens that can cause life-threatening anaphylactic reactions. In general, the use of biosensors in milk allergens detection offer many advantages in term of fast, repeatable, and highly sensitive approaches with great potential for full automation [28]. Furthermore, the biosensors (e.g., electrochemical and optical biosensors) have the potential to avoid the matrix treatment of via laborious techniques [29], supporting direct, real-time, and label-free detection (in the case of optical biosensors) for milk allergens detection. Furthermore, the use of the aptamers [27,30] and antibodies [31] as recognition element is emphasized to ensure the highly specific and sensitive performance of biosensors for the milk allergens detection. The commercially available biosensors are also summarized here as along with consideration of emerging challenges.

## 2. Analytical Standard Methods for the Milk Allergens Detection

In order to ensure the provision of food information to consumers, new legislation (i.e., the EU Food Information for Consumers Regulation 1169/2011) was introduced during December 2014 for all food businesses (i.e., deli counters, catering outlets, and bakeries). In this legislation, two different directives were incorporated for presentation and advertising the foodstuffs along with nutrition labelling. It was mandatory to provide allergen information on unpackaged food. The development of analytical techniques is of paramount importance to monitor the presence of allergens in both processed/unprocessed food keeping in view the public health implications and also labeling regulation. 

In recent years, several guidelines have been published over diagnosis as well as treatment of CMA; however, the major contribution is of the World Allergy Organization (WAO) Diagnosis and Rationale for Action against Cow’s Milk Allergy (DRACMA) [5,32]. The World Health Organization (WHO), the U.S. Food and Drug Administration (FDA), and other food safety management organizations are contributing in collaborative work for standardization of detection approaches for allergens and also to establish requirements for food labeling.

The detection techniques for food allergens differ in terms of basis of detection (biological or chemical), target analyte (DNA or proteins), phenomena of cross-reactivity of proteins, need for expertise, multitarget detection, and sensitivity to the specific allergens [25]. The thresholds of allergic reactions can be used as benchmark to specify the sensitivity limits of the sensors. The appropriate dose-distribution models have been reported to examine the minimal reactive doses to which maximum proportion of the people reacts [33]. Further, milk processing can also affect the several properties of the milk proteins such as increase in allergenicity due to pasteurization, decrease in antibody-binding capacity due to sterilization, and destruction of existing epitopes due to non-enzymatic glycation and denaturation [34].

The assessment of allergen labelling demands development of rapid, accurate, and cheap analytical techniques for sensing and quantification of milk allergens. The low concentration of allergens and also food matrices can be a problematic challenge in front of their detection. Most applied analytical approaches for milk allergens detection are: Protein-based methods and DNA-based methods [35,36]. These approaches differentiate in terms of the type of marker used for the allergen detection. In fact, DNA-based methods are based on specific DNA sequences that indicate the presence of the allergen. On the other side, protein-based methods are based on characteristic proteins (i.e., enzyme, antibody, etc.) as markers for detection of allergens. These are still most commonly exploited and available in different formats. These are based on antigen–antibody interactions including enzyme-linked immunosorbent assay (ELISA) [37,38], dipstick tests [39], and lateral flow devices (LFD) [40]. Furthermore, polymerase chain reaction (PCR) [41,42] and liquid chromatography-mass spectrometry (LC-MS) [26] platforms are mostly explored alternatives of antibody-based assays. Research efforts are directed for highly specific, sensitive, and rapid detection of allergens from the processed milk products [43,44,45]. Further, thermal and non-thermal processing of milk could make it hard to detect the proteins (i.e., responsible for milk allergy) using standard tests. However, the proteins of processed milk may still capable to cause runny eyes, itchy skin, wheezing, and other more serious symptoms [46]. In fact, few works reported the evaluation of immunoreactivity of pure whey and casein hydrolysates [47,48], but with limited application for detection of allergens from hydrolyzed milk proteins in various food matrices. Furthermore, heating and technological food processing could change the structure of target-protein or target-DNA structure compromising the final detection. Therefore, unambiguous identification and/or characterization of food allergens in different commercial products is essential to ensure the food safety. 

## 3. Biosensors in Detecting Food Allergens

According to the International Union of Pure and Applied Chemistry (IUPAC), the definition of a biosensor can be given as “a self-contained integrated device, which is capable of providing specific quantitative or semi-quantitative analytical information using a biological recognition element (biochemical receptor or bioreceptor), which is in direct spatial contact with a transducer. The transducer is used to convert (bio)chemical signal resulting from the interaction of the analyte with the bioreceptor into an electronic one. The intensity of signal is proportional to analyte concentration” [49]. Different biomolecules (i.e., antibody, enzyme, cells, oligonucleotide sequence, etc.) and biomimetic molecules (molecular imprinted polymer, aptamer, etc.) have been used as bioreceptors. 

Different types of devices have been reported depending on the modification transducer surface [50,51], bio-receptor employed (enzymes, antibodies, DNA) [52], immobilization strategy (covalent, non-covalent) [53,54], and detection mechanism (i.e., electrochemical, optical, etc.) [55]. The cross-reactivity of the biomolecules (i.e., immunoreactants and aptamers) to homologous proteins in milk of all ruminant species is the major factor that can affect the performance of biosensors. A similar protein composition can be seen between cow’s and buffalo’s milk; ewe’s and goat’s milk also have similar protein pattern to cow’s milk but with reduced α-casein fraction; camel’s milk possesses different pattern than cow’s milk in terms of several bands in caseins area and absence of β-LG [56]. Table 2 lists the allergens involved in cross-reactivity to milk proteins [7]. The cross-reactivity of antibodies was found between milk proteins (only caseins and β-LG) of cow and others (i.e., ewe, goat, and buffalo) but not with camel’s milk proteins [56]. In comparison to antibodies, the choice of specific aptamers can resolve the issue of cross-reactivity with other proteins of similar pattern [57]. In this section, both immunosensors and aptamer-based biosensors have been considered for the milk allergens detection (refer to Table 3).

For detection of milk allergens, the sample preparation is a very crucial step where extraction of the target analyte (i.e., specific DNA or proteins) without any kind of destruction or modification is carried out from the food matrices (like cheese, biscuits, cakes, etc.). For instance, sample preparation from cheese involves two different steps such as (i) mixing of cheese (0.05 g) with extraction buffer (1 mL) under shaking for 30 min at 60 °C and (ii) centrifugation for 10 min at 10,000 rpm to obtain the standard sample solution [58]. Extraction conditions such as temperature or pH and other chemical interferences may significantly affect the stability and/or integrity of the target [28]. In the case of the raw milk, the research efforts confirmed the detection of allergens with simple buffer-diluted milk samples, i.e., UHT, pasteurized, human, and raw bovine milk [59,60,61,62]. An appropriate dilution with buffer solution enables a good result in allergenic proteins detection. On the other hand, it is possible to separate the whey protein from the casein one through the acidification of raw skim milk. In fact, depending on the allergen to be determined, the acidification procedure, followed by thermal shock and centrifugation, can help to increase the biosensor selectivity [1,63,64,65,66].

**Table 2:** Details of milk proteins of other species associated with cross-reactivity phenomena (adapted with permission from ref. [7]).

Protein name	Buffalo	Goat	Sheep	Reindeer	Mare	Donkey	Mule	Camel	Pig
αS1-casein	Bub b 9	Cap h 9	Ovi a 9	Not found	Equ c 9	Not found	Not found	Cam d 9	Sus s 9
αS2-casein	Bub b 10	Cap h 10	Ovi a 10	Not found.0	Equ c 10	Not found	Not found	Cam d 10	Sus s 10
β-casein	Bub b 11	Cap h 11	Ovi a 11	Not found.0	Equ c 11	Not found	Not found	Cam d 11	Sus s 11
κ-casein	Bub b 12	Cap h 12	Ovi a 12	Not found	Equ c 12	Not found	Not found	Cam d 12	Sus s 12
α-Lactalbumin	Bub a 4	Cap h 4	Ovi a 4	Not found .0	Equ c ALA	Not found	Not found	Cam d 4	Sus s 4
β-Lactoglobulin	Bub a 5	Cap h 5	Ovi a 5	Ran t 5 .0	Equ c BLG	Equ as BLG	Equ mu BLG	Absent	Sus s 5
BSA	Not found	Cap h 6	Ovi a 6	Not found	Equ c 3 ^a^	Equ as 6	Not found	Not found	Sus s 1 ^a^

Bub b: *Bubalus bubalis*; Cap h: *Capra aegagrus hircus*; Ovi a: *Ovis aries*; Ran t: *Rangifer tarandus*; Equ c: *Equus caballus*; Equ as: *Equus asinus*; Equ mu: *Equus mulus*; Cam d: *Camelus dromedaries*; Sus s: *Sus scrofa domestica*; ^a^: Present in WHO/IUIS official list of allergens.

**Table 3:** Different biosensing platforms for detection of milk allergens.

S. No.	Type	Analyte	Biosensing Platform	Transduction Mechanism	Detection Limit	Linearity Range	Ref.
1	Immunosensor	β-lactoglobulin	Horseradish peroxidase labeled antibody immobilized activated carboxylic-modified magnetic beads/carbon	Amperometric	0.8 × 10^−3^ μg/mL	2.8–100 × 10^−3^ μg/mL	[67]
2		α-lactoglobulin	Horseradish peroxidase labeled antibody immobilized activated carboxylic-modified magnetic beads/carbon	Amperometric	11 × 10^−6^ μg/mL	37–5000 × 10^−6^ μg/mL	[62]
3		β-lactoglobulin	Anti-β-lactoglobulin antibody immobilized graphene/carbon	Electrochemical	0.85 × 10^−6^ μg/mL	1 × 10^−6^ to 100 × 10^−3^ μg/mL	[65]
4		β-lactoglobulin	Anti-β -lactoglobulin antibody immobilized gold sensor chip	Surface plasmon resonance	0.164 µg/mL	-	[68]
5		β-lactoglobulin	Anti-β-lactoglobulin antibody immobilized streptavidin coated quantum dots/functional copolymer, copoly (DMA-NAS) coated porous alumina membrane	Polarimetry	33.7 × 10^−3^ μg/mL	-	[69]
6		β-lactoglobulin	Double-antibody sandwich immunoassay	Surface plasmon resonance	5.54 × 10^−3^ μg/mL	5–40 × 10^−3^ μg/mL	[70]
7		α-lactalbumin	CdSe/ZnS quantum dots conjugated with monoclonal antibodies	Fluorescence-linked immunosorbent assay	0.1 × 10^−3^ μg/mL	0.1 to 1000 × 10^−3^ μg/mL	[71]
8		Casein and Immunoglobulin G	Integrated lab-on-a-membrane foldable device using Pb- and Cd-quantum dot tags	Electrochemical	0.04 μg/mL and 0.02 μg/mL	0–5 µg/mL and 0–2 µg/mL	[72]
9		Casein	Rat basophilic leukemia-immobilized graphene/carbon nanofiber/gelatin methacryloyl nanocomposites-based paper sensor	Electrochemical	3.2 × 10^-2^ μg/mL	0.1 and 3.2 μg/mL	[73]
10	Aptasensor	β-lactoglobulin	Aptamers immobilized graphene/carbon	Electrochemical	20 × 10^−6^ μg/mL	100 × 10^−6^ μg/mL to 100 × 10^−3^ μg/mL	[64]
11		β-lactoglobulin	Aptamer functionalized Fe_3_O_4_/cDNA conjugated carbon dots	Florescence	37 × 10^−6^ μg/mL	0.25 × 10^−3^ to 50 × 10^−3^ μg/mL	[74]
12		β-lactoglobulin	23-nucleotide aptamer-amphiphile	Enzyme linked apta-sorbent assay	10 nM	5 to 0.01 µM	[75]
13		β-lactoglobulin	Aptamer coupled poly(aniline-*co*-anthranilic acid)/graphite	Electrochemical	0.053 μg/mL	0.01 to 1.0 μg/mL	[76]
14		Lactoferrin	Bivalent aptamer linked to fluorescein isothiocyanate dye and silver decahedral nanoparticles	Fluorescence polarization	0.1 × 10^−3^ μg/mL	0.2 × 10^−3^ to 25 μg/mL	[77]
15		β-lactoglobulin	Microfluidic paper-based device with aptamer conjugated gold nanoparticles/graphene	Colorimetry	12.4 nM	25 nM to 1000 nM	[78]

### 3.1. Immunosensors

**Figure 1:**
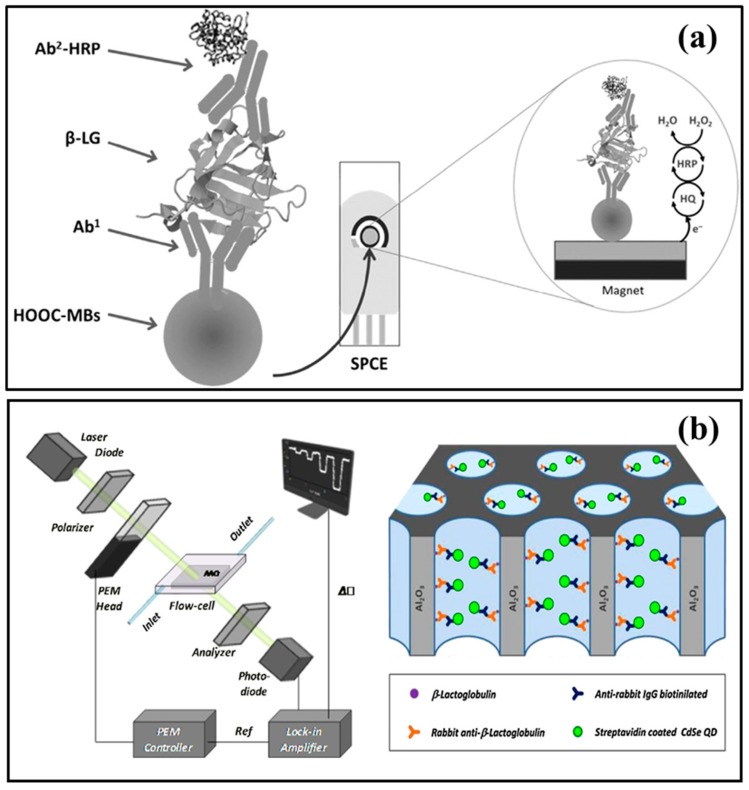
The most common immunosensors for milk allergens detection; (**a**) β-LG sandwich magneto immunosensor (reprinted with permission from ref. [67]) and (**b**) schematic layout of optical polarimetric platform to measure the phase retardation within the microporous alumina membranes carrying immunoassay for β-LG detection (reprinted with permission from ref. [69]).

In immunosensors, the immunochemical antibody–antigen (Ab–Ag) interactions are used as recognition elements. The sensitivities of the immunosensors is strongly dependent on the quality of antibody and transduction mechanism. Specifically, the capability of recombinant antibodies to be modified genetically offers several benefits in term of easy immobilization, excellent selectivity, and sensitivity. The biosensors combining the principle of solid phase immunoassay with magnetic beads (MBs) are ideal tools for immobilizing molecules like proteins (enzymes, antibodies, peptides, etc.) or nucleic acids. The immunoassays have been reported as versatile and powerful tool in several analytical and biotechnology applications [79,80]. Their use leads to improve the analytical performances of the biosensors (refer to Figure 1). Electrochemical magneto immunosensors based on sandwich approach are realized from Pingarrón’s group for the detection of β-LG and α-LA allergens [62,67]. The covalent immobilization of selected antibodies has been reported on carboxylic acid-functionalized magnetic beads (MBs) via amidic groups. The antibodies modified MBs were further incubated with sample solutions to capture the targets. After that, the affinity reaction with the secondary antibody (i.e., horseradish peroxidase-labeled) was carried out. The magnetically captured MBs over the surface of a screen-printed carbon electrode were utilized to examine the biorecognition event under amperometric measurements. The generated reduction current through hydroquinone HQ/H_2_O_2_ system was further measured and analyzed. The immusensors were able to detect both α-LA and β-LG retrieving a limit of detection (LOD) of 11.0 × 10^−6^ μg/mL [62] and 0.8 × 10^−3^ μg/mL [67], respectively. The magneto immunosensors were successfully applied in the milk samples analysis and their outcomes were further validated with commercial ELISA spectrophotometric kits. No doubt, the LOD of magnetoimmunosensor for β-LG was 10 times lower (i.e., 20 × 10^−6^ μg in 25 µL sample) than that of commercial ELISA kit (i.e., 195 × 10^−6^ μg), however the immunosensor showed remarkably faster detection process (i.e., about 60 min) than the kit (i.e., 4 h) [67]. Further, in case of α-LA detection, the immunosensor offered fast detection process (only 30 min) than ELISA kit [62]. Therefore, the immunosensors are highly efficient to detect the lower proteins contents (i.e., up to 10^−6^ μg) in the milk samples. In case of processed milk samples (e.g., pasteurized milk), the protein contents decrease than raw milk samples (i.e., 3–4 g/L) [81]. This decrease can be attributed to native structural changes in the proteins due to heat treatments. Moreover, irreversible structural changes in proteins due to processing can also alter their recognition by specific biomolecules.

A label-free voltammetric immunosensor using graphene-modified screen-printed electrode has been reported for detection of β-LG [65]. The aqueous acidic solution was used for derivatization of the electrode via electrochemical reduction of 4-nitrophenyl diazonium cations (i.e., in situ generated), which is further followed by the reduction of the nitro groups to amines. Further, the β-LG antibodies were covalently immobilized by the amine groups over glutaraldehyde-activated working electrode surface. A linear decrease in differential pulse voltammetry (DPV) reduction peak current of [Fe(CN)_6_]^3−/4−^ redox probe was observed with increased concentration of β-LG, which tends to the antigen-antibody complexes formation on the electrode surface. This immunosensor offered a LOD of 0.85 × 10^−6^ μg/mL and a linear detection range from 1 × 10^−6^ μg/mL to 100 × 10^−3^ μg/mL for β-LG standard solutions. Real samples (including cake, sweet biscuit, and cheese snacks) were also analyzed by the immunosensor, which confirmed the concentration of β-LG (i.e., 7.5, 7.47, and 87,330 μg/mL, respectively). The results of real samples were also validated with commercially ELISA method, i.e., 9.3, 7.7, and 92,300 μg/mL for cake, sweet biscuit, and cheese snacks, respectively.

Further, piezoelectric quartz crystal (QCM) sensors are also gaining considerable attention as competitive tool for characterization of biomolecular interactions as well as for bioanalytical assays [82,83]. In these sensors, the immobilization of bioreceptor is carried out over quartz crystal, which resonates due to the application of an external alternating electric field. Further, the biospecific reaction takes place between the two molecules interactive to each other, i.e., one free in solution and the other immobilized over the surface; this reaction can be examined in real time. Therefore, these biosensors are advantageous in terms of their simplicity of use, label free detection, and real-time monitoring. Ito et al. [68] demonstrated the analysis of β-LG using monoclonal antibodies and a flow-based QCM sensor that offered a LOD down to 1 × 10^−3^ μg/mL. However, QCM biosensor are facing some limitations in terms of sensitivity, specificity, and excessive interferences [84].

An immunosensor based on modified macroporous alumina membrane coupled with polarimetry was utilized to detect β-LG. Firstly, the membrane pore walls were coated with a functional copolymer, *N,N*-dimethylacrylamide-*N*-acryloyloxysuccinimide for the β-LG immobilization (Figure 1). Then, the immuno-assay proceeded to bind with the primary as well as secondary antibody cognates, i.e., rabbit anti-β-LG and anti-rabbit IgG, respectively. Further, quantum dots coated with streptavidin were used as enhancers for the refractive index signal. This immunosensor offered a 3.7 × 10^−3^ μg/mL (25 pM) noise floor for individual measurements, or formal assay with an LOD of 33.7 × 10^−3^ μg/mL (225 pM) [69]. 

The surface plasmon resonance (SPR) approach offers distinct benefits such as (i) flexibility and versatility and (ii) rapid, automated, and real-time analysis of diverse molecular interactions ranging from peptide–peptide and protein–protein to protein–cell interactions. Different optical biosensing approaches have been exploited for different applications in clinical, environmental, and food analysis [85,86,87,88,89]. SPR biosensors have also been developed for label-free and sensitive sensing of milk proteins [65,66]. Recently, an immunoassay based on SPR approach was reported for α-casein detection in rinse water samples at cleaning in place (CIP) of food manufacturing [90]. The SPR sensor showed good sensitivity with a LOD of 57.8 × 10^−3^ μg/mL, comparable to ELISA. Further, SPR biosensor based on immunoassay has also been developed for β-LG detection [68]. The optimized SPR biosensor was successfully examined for its detection performance, offering excellent sensitivity with 0.164 µg mL^−1^ LOD. An SPR method has also been reported for simultaneous and quantitative determination of different milk allergens such as α-LG, β-LG, lactoferrin, immunoglobulin G, and BSA in raw as well as processed milk with six samples per assay [66]. Further, an SPR immunosensor has been developed for the simultaneous detection of β-LG and Ara h1 allergens [70]. The monoclonal antibodies (mAbs) developed against β-LG and Ara h1 were immobilized on the biosensing chip to capture the proteins from the sample solutions. SPR biosensing platform offered detection limits for β-LG and Ara h1 as 5.54 and 0.77 × 10^−3^ μg/mL, respectively. More recent SPR immunosensor was reported for determination of both common β-LG variants in bovine milk by Indyk et al. [91]. In this case, the temporal variability in the β-LG content was studied in pasture-fed cows’ milk during both early lactation as well as production season.

Further, SPR assay was compared with (i) standard immunoassays (i.e., ELISA) for LF and IgG quantification in milk and whey and (ii) with HPLC for α-LG, β-LG, and BSA estimation in whey with correlation coefficients of R^2^ > 0.97 between these methods, except for IgG, which had R^2^ = 0.94 [66]. In addition, the assay was applied to analyze the contents of individual whey proteins in different dairy fluids such as liquid whey samples, commercial milk powders, skim milk, and whole milk. Moreover, surface plasmon resonance imaging (SPRi) was applied for anti-bovine IgG sensing in both untreated human serum as well as milk, ranging from 0.1 to 1.0 × 10^6^ μg/L. The LOD was found 0.11 × 10^6^ μg/L in diluted samples [92].

Further, microcantilever (MC)-based biosensing platforms have been applied for detection of various clinical targets [93]. However, only limited work is reported on the application of MC resonator array for determination of desired biomolecular analytes in the foodstuff. An immunosensor based on MC resonator arrays is reported by Ricciardi et al. [94] for β-LG detection. The developed sandwich immunoassay offered better LOD (0.04 × 10^3^ μg/mL) than commercial ELISA tests (0.19 × 10^3^ μg/mL) with the use of same biorecognition element [71].

Other strategies for sensitive voltammetric analysis of allergens involve the coupling of bioreceptors with novel features (i.e., coding and amplification) of inorganic nanocrystals in order to achieve a highly selective, sensitive, and simultaneous sensing of multiple protein targets. A competitive immunosensor was proposed by Kokkinos et al. [72] for bovine immunoglobulin G (bIgG) and bovine casein (CN) detection. The electrochemical devices are realized by screen-printed cell (SPC) with nylon membranes, symmetrically located on each side of SPC. Further enhancing the assay sensitivity, the graphite screen-printed electrode was modified with a bismuth citrate. The competitive immunoassay was performed using biotinylated antibodies labeled with Cd- and Pb-based quantum dots conjugated with streptavidin. Upon dissolving these labels, the release of Cd(II) and Pb(II) was observed in the assay zones. After that, the two assay zones were folded over and brought in contact with electrochemical cell where anodic stripping voltammetric (ASV) was performed. The bismuth citrate reduces during the preconcentration step. Duplex ASV-QDs-based detection of both bIgG and CN was performed in milk samples yielding an LOD of 0.02 μg/mL and 0.04 μg/mL, respectively. Further, the immunosensors have also been reported for reliable determination of IgGs from adulterated milk with colostrum or milk from other animals (i.e., goats, cows, and sheep). The electrochemical bioplatform based on antibody-conjugated magnetic beads offered excellent LODs of 0.66, 0.74, and 0.82 × 10^−3^ μg/mL for caprine, bovine, and ovine IgGs, respectively [95]. The obtained detection limits are much lower than that of ELISA methodologies (i.e., between 0.7 and 1.95 × 10^−3^ μg/mL) for IgG detection. This bioplatform was successfully applied to different types of samples (i.e., diluted milk samples) such as (i) raw, pasteurized, and colostrum milk samples and (ii) milk samples from different animals and provided fast results, i.e., within 30 min. The magnetic beads can offer several advantages in biosensing platforms such as easy functionalization, large surface area, improved sensitivity, matrix effect minimization, rapid assay kinetics, and easy control over location/transport with the help of a magnetic field [96,97]. 

Due to recent advancements in smartphone and realization of new apps, many researchers focused their attention on the use of ‘smart devices’ in bioanalytical applications [98,99]. Recent research publications have successfully explored the feasibility of such smart devices for sensing of a wide range of targets in biological fluids [100,101,102]. The incorporation of smartphone technology with immunosensors have been explored for detection of numerous food allergens, however limited work is reported on milk allergens detection [103,104,105,106]. An amperometric biosensor based on an inhibitory immunoassay for detection of β-casein was reported by Molinari et al. [107]. For detection, eight electrochemical cells were integrated into a portable potentiostat, which is controlled by a smartphone via Bluetooth. The determination of β-casein in the 0–10 ppm range was performed and an LOD of 0.173 × 10^3^ μg/mL was achieved.

The mast cells have a natural ability to determine the presence of the food allergens and further produce proinflammatory responses. These offer an accurate and stable strategy mimicking the physiological conditions [108]. A new paper-based analytical device has been reported for the sensitive detection of casein [73]. The sensor utilized casein antibody-sensitized mast cells that were immobilized on graphene/carbon nanofiber/gelatin methacryloyl nanocomposite material-based electrode. The developed paper-based sensor using cells offered a range of linearity from 0.1 to 1 μg/mL of casein detection with an LOD of 3.2 × 10^−2^ μg/mL.

To determine the fraudulent substitution of goat milk along with its derivatives in bovine milk, a label-free immunosensor for real-time detection of k-casein based on broad-band Mach–Zehnder interferometry was proposed by Angelopoulou et al. [109]. The LOD of the assay was observed to be 0.04% (*v*/*v*) and the working range from 0.1 to 1% (*v*/*v*) of bovine in goat milk [110]. Other immunosensors include a surface-enhanced Raman scattering-based lateral flow strips for sensing of milk allergens [111]. Besides these advancements in immunosensors for faster allergen detection, all techniques based on antibodies (whether ELISA or immunosensors) suffer from difficulties in the analysis of processed food. In the case of processed food, the treatment and the processing of food matrix led to denaturation, hydrolysis, and conformational changes in proteins, due to which they cannot be recognized by antibodies properly [112]. Moreover, the quality of antibodies is also crucial for development of ultra-sensitive immunosensors.

### 3.2. Aptasensors

Aptasensor are affinity biosensors, in which aptamers are immobilized at the electrode surface and are used as the bioreceptor to selectively bind the target analyte. The binding event leads to change of electrical or optical analytical signal at transducer surface and therefore could be easily monitored. Nowadays, aptamers are replacing other biorecognition elements, especially antibodies with similar functionality due to enormous benefits in terms of more stability, easy and cheaper production, lasting longer, and availability of more modification choices [113,114,115,116,117]. Although the use of antibodies as biological reagents is very common, there, however, are several issues associated with antibodies such as ethical issues in their production inside animals, time-consuming process, and high-cost manufacturing. In spite of antibodies, aptamers are more stable over a good range of temperature and can be produced by in vitro chemical processes. Further, some aptamers have high specificity and affinity for target binding than antibodies. Moreover, they can be easily modified with new functional groups due to the possibility of reversible denaturation [118]. 

Over the past few decades, the significant work can be seen on production of aptamers against various targets via systematic evolution of ligands by exponential enrichment (SELEX) [30,114]. Especially for milk allergens, silver decahedral nanoparticles and fluorescence activated cell sorting (AgNPsFACS)-based SELEX has been reported for highly sensitive and specific screening of aptamers against respective milk allergens [119]. Some aptamers already screen targeting milk proteins (refer to Table 4).

**Figure 2:**
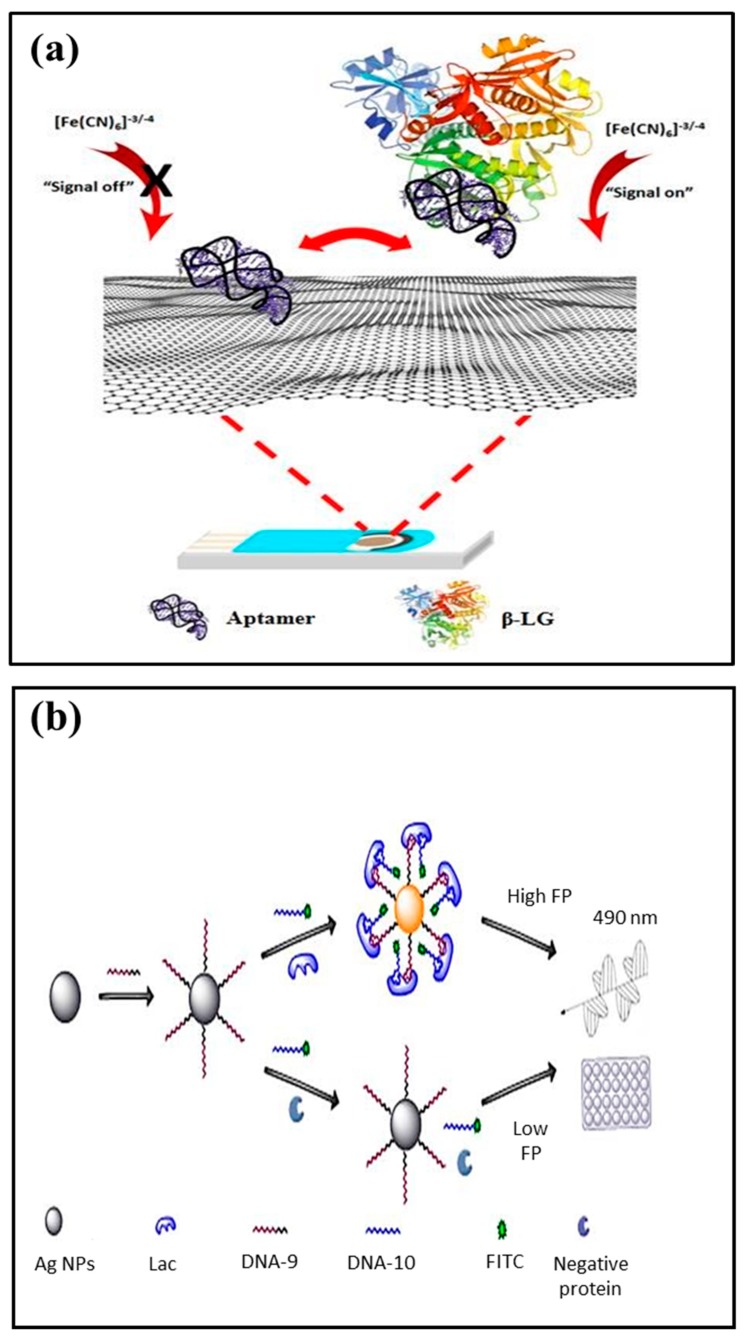
The most common aptasensors for milk allergens detection; (**a**) electrochemical detection of β-LG using aptamer-functionalized graphene screen-printed electrodes (reprinted with permission from ref. [64]) and (**b**) bivalent aptasensor using sliver decahedral nanoparticles for enhanced detection of lactoferrin in milk (reprinted with permission from ref. [77]).

Among food allergens detectable by aptasensors, only a few examples of aptasensors are reported in literature to detect different milk allergens (refer to Figure 2) [121,122]. An electrochemical disposable platform based on aptamer-coupled ploy(aniline-co-anthranilic acid) (PANI/PAA) deposited graphite electrode offered effective detection of β-LG via differential pulse voltammetry [76]. The LOD was found 0.053 μg/mL in β-LG-spiked milk samples. Eissa and Zourob [64] selected various aptamer sequences with high selectivity and specificity to both β-LG A and B with dissociation constants (Kds) of 82 and 80 nM. The aptamer sequence selected for β-LG was immobilized on graphene-modified screen-printed carbon electrodes. The binding between the aptamer and the target was monitored via the square wave voltammetry (SWV) where changes in the reduction peak signal of ferrocyanide/ferricyanide redox couple can be observed due to the negatively charged aptamer’s removal from the electrode surface upon target protein binding [64]. 

A fluorometric aptamer assay for detecting β-LG based on the use of magnetic nanoparticles and carbon dots as a signal indicator has been developed by Shi et al. [74]. The assay was based on the hybridization between aptamer, immobilization on magnetic nanoparticles, and the complementary oligonucleotide sequence labeled with carbon dots. In the presence of β-LG, the aptamer preferentially binds to the protein, and this binding leads to a partial release of the complementary sequence into the solution. After magnetic separation of the nanoparticles, the supernatant of the solution contained the released carbon dots, which were further quantified by fluorometry. The β-LG was measured in the 0.25 × 10^−3^ to 50 × 10^−3^ μg/mL range and an LOD of a 37 × 10^−6^ μg/mL was achieved. This approach was successfully examined for sensing of β-LG in hypoallergenic formulations.

Further, highly specific β-LG-23 aptamer binding affinity to β-LG has been reported via enzyme linked apta-sorbent assay [75]. The proposed biosensor offered effective detection of β-LG with a LOD of 0.18 μg/mL or 10 nM within 20 min. The results are comparable to commercially available ELISA kit (provided by Crystal Chem) that has an LOD of 0.31 × 10^3^ μg/mL with a 3 h assay time. 

A novel bivalent aptasensor based on fluorescence polarization has been reported for lactoferrin detection by Chen et al. [77]. The fluorescence polarization is an effective technique where high sensitivity to the rate of rotation can be achieved by target-induced conformational or structural changes. In this aptasensor, two split aptamers were verified and modified to link with fluorescein isothiocyanate dye molecules and silver decahedral nanoparticles (working as enhancer), respectively (Figure 2). The aptamers, upon binding with lactoferrin, form a split aptamers-target complex that narrow down the distance between the dye and silver nanoparticles. In this case, mass-augment and also an enhanced fluorescence effect can be observed due to silver decahedral nanoparticles. In comparison to traditional aptamer-based homogeneous assays, the bivalent aptasensor offered a sensitivity three times higher with a LOD of 1.25 pM for lactoferrin in milk powder.

Further, paper-based microfluidic devices are gaining considerable attention for point-of-care (POC) testing [123]. These devices are based on natural capillary action of cellulose substrate in order to perform the diagnostic test. Tah et al. [78] reported the conjugation of aptamers to gold nanoparticles and the utilized graphene oxide with a paper-based microfluidic device. Here, the functionalized gold nanoparticles offered a bridging effect between different layers of graphene oxide via pi-pi stacking that can be due to the gold nanoparticles-conjugated ssDNA aptamer. The microfluidic platform offered high sensitivity for colorimetric detection of β-LG with a LOD of 12.4 nM. The microfluidic platform has the potential for low-cost, rapid, and accurate POC device for real-time detection of milk allergens. Beyond these, several works have been reported on aptamer-based detection of pesticide residues [124], antibiotics [125,126,127], and others [128] from milk samples. However, there is still need to screen aptamers for other milk allergens, e.g., casein [55].

## 4. Commercially Available Biosensors

The biggest challenge for milk allergens detection is the lack of highly sensitive, low-cost, and user-friendly detection kits in the market. In order to ensure the milk safety from toxic substances and allergens, the user-friendly detection kits should be commercially available for use by individual consumers, food manufacturers, and food safety organizations. In the market, the available major milk allergens kits are as (i) ELISA kits such as Beta-lactoglobulin ELISA-Type II by Crystal Chem [129], SENSI*Spec* Beta-Lactoglobulin ELISA kit by Eurofins Technologies [130], RIDASCREEN^®^FAST Milk by R Biopharma [131], etc. and (ii) lateral flow-based immunochromatic test kits such as AgraStrip^®^ Total Milk by Romer Labs [132], Neogen’s Reveal 3-D [133], Lateral Flow Milch/Milk by R-Biopharm AG [134], AlerTox Sticks Total Milk by Emport LLC [135], Charm Aller-ROSA Milk Test [136], etc. Several activities have been initiated for commercialization of biosensors with capabilities for detection of multiplexed allergens. For example, SensoGenic Pvt. Ltd., Israel is developing portable and digital diagnosis-based biosensors for allergens detection from food matrices (i.e., peanuts, milk, tree nuts, soy, fish, eggs, shellfish, and wheat) [137]. Currently, the transfer of biosensors from lab scale to commercial market is hampered by several limitations in terms of stability of biological receptors and costly development of biological sensing layers. Research activities for artificial receptors (such as aptamers) have been initiated but still only a limited number of aptamers have been screened for target analytes. Further, the incorporation of nanomaterials in biosensors has led to improved selectivity, sensitivity, high sample throughput, rapid analysis, and better efficiency in analysis from complex sample matrices than conventional analytical approaches. The combination of biomolecules with nanomaterials with novel characteristics showed immense potential for development of real-time, miniaturized, portable, cost-effective, and rapid biosensors possessing capabilities for detection of multiple allergens. These ongoing advancements in highly sensitive, accurate, low-cost, and easy to use biosensors are significant for quality control as well as food safety regulation. These biosensors are expected to become available in market from laboratory research in coming years.

## 5. Conclusions and Future Prospects

In order to protect the sensitized individuals to cow’ milk allergens, the development of highly sensitive, selective, and accurate detection methods is necessary. Recently, biosensors (i.e., immunosensors and aptasensors) in combination with novel nanomaterials and sensing platform fabrication are offering a cheaper, facile, rapid, and multiplex detection of milk allergens in comparison to conventional techniques like ELISA, LC-MS, and real-time PCR. Here, we reviewed the considerable developments in both immunosensors and aptasensors for milk allergens detection. Besides significant potential of immunosensors in milk allergens detection, the major challenges in terms of instability and high cost of antibodies still need to be resolved. Although the highly specific reactivity of aptamers can resolve the issue of cross reactivity of detectors, the effect of variability of allergens presentation from different species on detection approach needs to be examined. The aptasensors for detection of milk allergens are in preliminary stage of development and still possess a long way to go for more acceptable diagnostic tool with high stability. The ongoing research for the development of new biosensors or biosensing kits is of paramount importance to reduce the gap between research at lab scale and the commercial applications.

## Figures and Tables

**Table 1:** Cow’s milk allergenic proteins and their characteristics according to the World Health Organization and International Union of Immunological Societies (IUIS) allergen nomenclature [8,12].

	Protein Name	Allergen Nomenclature	Conc. in Milk (g L^−1^)	Isoelectric Point	Number of Amino Acids/Molecules
Casein proteins (80%) (≈5 g L^−1^)	αS1-casein	Bos d 9	12.0–15.0	4.9–5.0	199
	αS2-casein	Bos d 10	3.0–4.0	5.2–5.4	207
	β-casein	Bos d 11	9.0–11.0	5.1–5.4	209
	κ-casein	Bos d 12	3.0–4.0	5.4–5.6	169
Whey Proteins (20%) (≈30 g L^−1^)	α-Lactalbumin	Bos d 4	1.0–1.5	4.8	123
	β-Lactoglobulin	Bos d 5	3.0–4.0	5.3	162
	BSA	Bos d 6	0.1–0.4	4.9–5.1	582
	Immunoglobulins	Bos d 7	0.6–1.0		

**Table 4:** Screened aptamer sequences with binding affinity for milk allergens.

S. No.	Aptamer Sequence	Affinity Constant, K_d_ (nM)	Targeted Milk Allergen	Ref.
1	CGACGATCGGACCGCAGTACCCACCCACCAGCCCCAACATCATGCCCATCCGTGTGTG	82 ± 30 and 80 ± 26	β-lactoglobulin A and B	[64]
2	5′-GGGGTTGGGGTGTGGGGTTGGGG/3AmMO/-3′	22 ± 2	β-lactoglobulin	[75]
3	5′-FITC-AGGCAGGACACCGTAACCGGTGCATCTATGGCTACTAGCTCTTCCTGCCT-3′	28.78 ± 7.20	lactoferrin	[77]
4	ATA CCA GCT TAT TCA ATT CGA CGA TCG GAC CGC AGT ACC CAC CCA CCA GCC CCA ACA TCA TGC CCA TCC GTG TGT GAG ATA GTA AGT GCA ATC T	--	β-lactoglobulin	[78]
5	CGGTGCATCTATGGCTACTAGCTTTTCCTGCCTATACTAC	1.04 ± 0.50	lactoferrin	[120]
